# Diamide insecticide resistance in transgenic *Drosophila* and Sf9‐cells expressing a full‐length diamondback moth ryanodine receptor carrying an I4790M mutation

**DOI:** 10.1002/ps.6730

**Published:** 2021-12-03

**Authors:** Ewan Richardson, Rafael A Homem, Bartlomiej J Troczka, Christopher H George, Ulrich Ebbinghaus‐Kintscher, Martin S Williamson, Ralf Nauen, TG Emyr Davies

**Affiliations:** ^1^ Department of Biointeractions and Crop Protection Rothamsted Research Harpenden UK; ^2^ College of Life and Environmental Sciences, Biosciences University of Exeter, Penryn Campus Penryn UK; ^3^ Institute of Life Sciences, Swansea University Medical School Swansea UK; ^4^ Bayer AG, Crop Science Division, R&D Monheim Germany

**Keywords:** diamide insecticides, chlorantraniliprole, flubendiamide, *Plutella xylostella*, diamondback moth, insecticide resistance

## Abstract

**BACKGROUND:**

Resistance to diamide insecticides in Lepidoptera is known to be caused primarily by amino acid changes on the ryanodine receptor (RyR). Recently, two new target site mutations, G4946V and I4790M, have emerged in populations of diamondback moth, *Plutella xylostella*, as well as in other lepidopteran species, and both mutations have been shown empirically to decrease diamide efficacy. Here, we quantify the impact of the I4790M mutation on diamide activation of the receptor, as compared to alterations at the G4946 locus.

**RESULTS:**

I4790M when introduced into *P. xylostella RyR* expressed in an insect‐derived Sf9 cell line was found to mediate just a ten‐fold reduction in chlorantraniliprole efficacy (compared to 104‐ and 146‐fold reductions for the G4946E and G4946V variants, respectively), whilst in the field its presence is associated with a ≥150‐fold reduction. I4790M‐mediated resistance to flubendiamide was estimated to be >24‐fold. When the entire coding sequence of *P. xylostella RyR* was integrated into *Drosophila melanogaster*, the I4790M variant conferred ~4.4‐fold resistance to chlorantraniliprole and 22‐fold resistance to flubendiamide in the 3rd instar larvae, confirming that it imparts only a moderate level of resistance to diamide insecticides. Although the I4790M substitution appears to bear no fitness costs in terms of the flies' reproductive capacity, when assessed in a noncompetitive environment, it does, however, have potentially major impacts on mobility at both the larval and adult stages.

**CONCLUSIONS:**

I4790M imparts only a moderate level of resistance to diamide insecticides and potentially confers significant fitness costs to the insect.

## Graphical abstract

The I4790M mutation on PxRyR imparts only a moderate level of resistance to diamide insecticides (as compared to G4946E or G4946V) and potentially confers significant fitness costs to the insect.

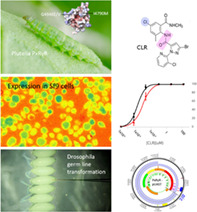

© 2021 The Authors. *Pest Management Science* published by John Wiley & Sons Ltd on behalf of Society of Chemical Industry.

## 1 INTRODUCTION

Insecticide resistance, or the reduction in control efficacy of a synthetic insecticidal compound, is caused by repeat application of the compound over multiple generations of an insect population. Genes for resistance mechanisms, already present within the population, increase in frequency within the population due to the relative adaptive fitness of their bearers. A stark illustration of the potential for the rapid emergence of such resistance in a field population can be seen in the case of diamide insecticides. Just 18 months after their market introduction, resistance to diamide insecticides emerged in the Philippines in a population of diamondback moth (*Plutella xylostella)* and was quickly followed by further episodes in nearby regions of Southeast Asia.^1^ The resistance resulted from the rapid proliferation of a target‐site polymorphism, G4946E, located close to the C‐terminus of the insect ryanodine receptor (RyR).^1^ In the subsequent five years, numerous studies were published reporting the presence of this mutation in resistant *P. xylostella* populations from locations across the globe (reviewed comprehensively in Richardson *et al*.^2^). An alternative substitution, G4946V, also has recently emerged, characterized in *P. xylostella* populations collected in China.^3^ Additionally, mutation at this G4946 residue has been implicated in diamide resistance in several other lepidopteran pest species.^4^, ^5^ An overview of the past decade of scientific literature^2^ suggests that, in the presence of diamide selection, survivorship of G4946E/V variants over wild‐type (WT) frequently is increased >3000‐fold.

A decade on from those first reports of diamide resistance in *P. xylostella*, resistance now is known to have evolved independently in at least nine lepidopteran pest species. Diamide resistance therefore is no longer exclusive to *P. xylostella* but also is now present in diverse populations of *Tuta absoluta*, *Spodoptera frugiperda*, *Spodoptera exigua* and *Chilo suppressalis*. Subsequent expansion of some of these species out of their native range has exacerbated the spread of diamide resistance, with several species making the transition from regional to global pest status. Unsurprisingly, this diversification is coupled to the emergence of new genotypic mechanisms. One *RyR* amino acid residue in particular, I4790M (*P. xylostella* numbering), has been implicated in diamide resistance within all of the species listed above (PxRyR, *P. xylostella RyR*). Detected in resistant lepidopteran pests from China,^6^, ^7^, ^8^, ^9^ Brazil,^10^ Europe^11^ and Korea,^12^ this residue is developing a global importance to rival that of the G4946 locus. Investigations into I4790M‐mediated resistance therefore were the focus in this study.

## 2 MATERIALS AND METHODS

### 2.1 Chemicals

Chemicals used for the preparation of bacterial media were purchased from Sigma (Sigma‐Aldrich, St Louis, MO, USA). Analytical grade DMSO (purity ≥99%) used for dilution of all active compounds was obtained from Sigma. Technical grade flubendiamide sulfoxide (FLB) and chlorantraniliprole (CLR) (purity>98%) were provided in‐house (Bayer CropScience, Monheim, Germany) or purchased as analytical standard from Fluka Chemicals (Buchs, Switzerland), respectively. Analytical grade caffeine was purchased from ReagentPlus® (Sigma).

### 2.2 Mutagenesis of PxRyR

Construction of the pIZ‐WT‐PxRyR/V5‐His plasmid used in this study was as described in Troczka *et al*.^13^ For the preparation of the PxRyR‐G4946E variant, glutamic acid (E) was introduced into ‘fragment F4’ of the open reading frame (ORF) before re‐assembly of the full‐length ORF, as described in Troczka *et al*.^13^ The individual single nucleotide polymorphism (SNP) changes I4790M and G4946V likewise were incorporated into fragment F4 using the methods described previously^13^ and primers listed in Supporting Information, Table S1. However, in both cases a modification was made to the previously described protocol to facilitate fragment re‐assembly (further details are available in Methods S1). Transformed, purified PxRyR assemblies were validated for completeness via diagnostic digestion, complete amplification, and complete Sanger sequencing (Eurofins Genomics UK Ltd, Wolverhampton, UK).

### 2.3 Sf9 transfection protocol

*Spodoptera frugiperda* Sf9 cells (Life Technologies, Carlsbad, CA, USA) were grown at 27°C in S900™ II SFM (Gibco – Thermo Fischer Scientific, Waltham, MA, USA) in 30‐mL suspension cultures supplemented with 0.6% FBS (Gibco – Thermo Fisher Scientific). Transfection of cells with the pIZ‐PxRyR/V5‐His expression plasmid and Cellfectin™ (Thermo Fisher Scientific) was performed according to the manufacturer's instructions. Glass coverslips (1 cm^2^ diameter) coated with Poly‐l‐lysine (Sigma) were placed in a four‐well plate. Each well then was filled with 0.5 mL Sf‐900™ II medium and each coverslip was seeded with approximately 150 000 cells, at a density of 800 cells mm^–2^, to produce a ≈90% confluent monolayer. Cells were allowed to attach to the coverslips for 16 h and then were transfected. Transfection solution was composed of 3.25 μg pIZ‐PxRyR/V5‐His plasmid DNA dissolved in water; 4.5 μL PLUS™ enhancer reagent (Thermo Fisher Scientific); 20 μL Cellfectin™ (Thermo Fisher Scientific); per 1 mL of fresh Sf900™ II SFM. The Cellfectin and DNA:PLUS solutions were individually mixed and incubated for 5 min, before being combined and incubated for a further 30 min. Cells were removed from their media and washed twice, before addition of transfection solution. Transfection incubations proceeded for 4 h, before the cells were washed and returned to 30% conditioned SF900™ II SFM, with 0.6% FBS. Post‐transfection, cells were incubated at 27°C for 40–52 h.

### 2.4 Calcium imaging and data collection

Fura 2‐AM dye (Life Technologies) was used for monitoring calcium release in Sf9 cells transfected with recombinant PxRyR. At 48 h post‐transfection cells were loaded with Fura 2‐AM calcium‐sensitive dye. Cells on coverslips in four‐well plates first were put into 500 μL fresh SF‐900 II SFM and then 2 μL dye stock solution (1 mmol L^–1^) was added (to generate a final concentration of 4 μmol L^–1^). Cells were left to incubate at 27 °C for 45–60 min, followed by three washes with 500 μL fresh unsupplemented Sf‐900 II SFM. Before imaging, coverslips with Fura‐2‐AM loaded cells were placed in standard Ringer's solution containing 2 mmol L^–1^ [Ca^2+^] (CaCl_2_). All experiments were carried out in an air‐conditioned room maintained at ≈25 °C. Data collection for all the calcium imaging studies took place on a Ratiometric Imaging Perfusion system (RIPS), utilizing an Axio Vert.A1 microscope with a LD Plan‐Neofluar x10/0.4 lens (Zeiss, Oberkochen, Germany), measuring the ratio of excitation at 340/380 nm (calcium free/calcium bound indicator) every 180 ms and capturing emission at 510 nm (±20–30 nm). Cells on the coverslip were placed into a perfusion chamber of ≈0.5 mL volume mounted on the microscope stage. Continuous unidirectional flow of Ringer's medium through the bath was driven by a peristaltic pump, allowing for a constant fluid exchange. Caffeine and diamide agonist test solutions were applied using 3‐s bursts via a metal U‐tube. Fluid dynamics were measured using a solution of red amaranth dye, diluted 1:20 in Ringer's. The perfusion flow rate was 49 μL s^−1^. Experiments on cells consisted of multiple agonist applications, with the order and timing of applications dependent on the experimental aims. Recordings began at *T* = 0 s, with application of 10 mmol L^–1^ caffeine at 7–10s, followed by a 140 s delay, during which period caffeine‐responsive cells in the field‐of‐view (FOV) were identified (i.e. those cells exhibiting a large biphasic Ca^2+^ event following the application of caffeine). During concentration–response experiments a single caffeine application was followed by a single diamide application at 150 s. Measurements were taken for all four PxRyR constructs in a single day, in order to minimize methodological variation, with data being acquired from 6 to 25 cells per coverslip. Experiments were recorded using VisiView® (Visitron Systems, Puchheim, Germany) software. Raw video capture on the software was used to identify caffeine‐responsive cells. Outputted numerical pixel intensity data were analyzed using excel (Microsoft, Redmond, WA, USA) and sigmaplot v12 (Systat Software, Chicago, IL, USA).

### 2.5 Agonist diluent and background fluorescence

Owing to their low solubility in water, stock PxRyR agonists used in this study initially were dissolved in DMSO and then further diluted into Ringer's medium at a dilution factor of 1:100. As the 1% DMSO was found to cause a gradual rise in baseline Ca^2+^ in some Sf9 cells (both transfected and nontransfected), the fluorescence amplitude of cells exhibiting no change in fluorescence in response to caffeine was measured during each diamide measurement, and then subtracted from the fluorescence of each responding cell in order to adjust for any DMSO‐mediated fluorescence. Likewise, changes in background fluorescence due to application of agonist frequently occur in nonratiometric calcium imaging, either due to changes in solution viscosity or due to poor dispersal of agonist in the media. In this present case, use of a ratiometric dye compensated for the issue of background disturbance, and the effects occur equally at both ratiometric wavelengths, and therefore cancel each other out. Additionally, pluronic F68 (Gibco – Thermo Fischer Scientific) was added to the final solutions of all agonists (including caffeine) at 0.003% concentration, in order to aid the solubility of the diamide compounds.^14^

### 2.6 Calculating proportional normalized response (PNR) of individual cells to diamide insecticides

Responses of individual cells to the application of caffeine, and then diamide compounds, were recorded. Diamide response amplitude was normalized to the prior caffeine response to create a response ratio, which then was normalized against the maximal caffeine responses to establish the proportional normalized response (PNR). In brief, to calculate the PNR, raw data were normalized using the equation: *R*/*R*_0_, where *R* is the fluorescence ratio value recorded for an individual cell upon each individual time point and *R*_0_ is an average fluorescence ratio calculated over the first 5 s before addition of the agonist. The maximum response amplitude is taken as the maximum fluorescence signal outputted by the cell across all time frames. Final amplitude data are presented as a mean value and the standard deviation (SD) of the mean. In all of the concentration–response plots, response data are expressed as a percentage of the highest response registered. The magnitude of Ca^2+^ release occurring in response to diamide addition was normalized to the initial caffeine‐evoked Ca^2+^ release in the same cell (10 mmol L^–1^ caffeine application occurred 150 s before diamide application). A full description of the data analysis pipeline employed is provided in Methods S2. A WT‐PxRyR EC_50_ of 0.015 μmol L^–1^ for CLR and 0.27 μmol L^–1^ for FLB (Fig. S1) were comparable to those generated by previous authors^13^ (EC_50_s = 0.017 μmol L^–1^ for CLR; 0.25 μmol L^–1^ for FLB).

### 2.7 *Drosophila melanogaster* rearing

Strains of *D. melanogaster* were maintained in standard 25 × 95mm polystyrene vials (Genesee Scientific, San Diego, CA, USA) with 5 mL standard fly food (Nutri‐Fly® Bloomington formulation). Fly stocks were kept at 19 °C, in a 12 h:12 h, light:dark photoperiod, at 65% relative humidity (RH), and transferred to fresh vials every four weeks. Virgin female *D. melanogaster* for crosses were collected within 8 h of emergence.

### 2.8 Transfer of recombinant PxRyR from pIZ/V5‐His to create pUAST‐PxRyR expression vector

The PxRyR cassette (containing the C‐terminal fragment of PxRyR) was transferred between the two insect expression vectors used in this study, pIZ/V5‐His and pUAST, via a *Kpn*I digestion strategy. The C‐terminal *RyR* fragment is flanked by two *Kpn*I sites, 1798 bp upstream and 1 bp downstream, respectively. Simultaneous digestion of pIZ‐PxR/V5‐His and pUAST‐WT‐PxRyR with *Kpn*I, and electrophoresis in 0.5% agarose (60 V, 1 h) gave bands of 8151 bp, for the C‐terminal fragment containing the mutation and either 10 020 bp for the pIZ‐RyR/V5‐His or 15 729 bp for the pUAST‐RyR (Fig. S2). Purification and ligation of the required fragment with the vector backbone, followed by *Escherichia coli* transformation, then was employed to generate the final pUAST‐PxRyR construct.

### 2.9 Generation of the injection line

An attP integration strain RyR16.attP carrying a truncated *RyR* allele^15^ was generated by replacing chromosome 2 from strain y[1] M{vas‐int.Dm}ZH‐2A w[*]; M{3xP3‐RFP.attP}ZH‐86Fb by chromosome 2 from strain y1] w[*]; RyR [16]/CyO, y[+]. Both strains were acquired from the Bloomington Drosophila Stock Centre (reference nos. 24749 and 6812, respectively). A crossing scheme detailing how this strain was generated is shown in Fig. S3. The RyR16.attP strain expresses the φC31 integrase under the control of the *vasa* promoter, allowing for efficient transformation just within the germline cells. Upon the second chromosome, it carries a null‐functional truncated *RyR* mutated allele^15^ over a balancer, and upon the third chromosome, an attP integration site, position 86F8, where the inserted PxRyR sequence will be integrated.

### 2.10 Generation of UAS‐PxRyR transgenic Drosophila lines

For UAS‐PxRyR integration, the φC31 recombination system was used, whereby integrase catalyses recombination between an attB site present in the pUAST‐PxRyR vector and an attP site present in the genome of the RyR16.attP strain in a nonreversible manner, integrating the entire vector into the fly genome (following Bischof *et al*.^16^).

Approximately 150 female and 150 male flies of the RyR16.attP strain were transferred to a cage containing an egg‐laying plate [FlyStuff grape agar mix (Genesee Scientific) streaked with yeast paste (Red Star; Lessaffre Yeast Corporation, Milwaukee, WI, USA)]. The adult flies were added to the cage two days before embryo injection and left at 25 °C to acclimate, and the food was changed two to three times a day. On the day of injection, fresh eggs were collected every 30 min to ensure that injection was carried out using embryos in which blastoderm cells had not formed. Once the embryos were injected (injection protocol as described in Methods S3), the coverslip was prepared for embryo incubation by draining the halocarbon oil, rinsing with 70% ethanol, rinsing with water and gently drying. The coverslip then was slotted into a food vial with 5 mL Nutri‐Flyfood (Genesee Scientific) supplemented with 5–10 grains of dry yeast (Red Star, Lessaffre Yeast Corporation). One coverslip was placed per vial, ensuring the embryos remained close, to but not immersed in, the food and the approximate number of intact embryos recorded on the vial. Embryo vials were incubated at 25 °C at 90–100% relative humidity and transferred to 50–70% relative humidity at 48 h. Pupae were transferred to new vials and F_0_ virgin females and males were collected and isolated as they emerged.

### 2.11 Screening of UAS‐PxRyR flies

Two fly strains were generated; one integrated with the WT‐PxRyR sequence, a second containing PxRyR with a I4790M substitution (identified in *P. xylostella* and *T. absoluta*). Surviving embryos (F_0_) were reared at 25 °C to adulthood and backcrossed with noninjected flies of RyR16.AttP. F_1_s were scored for the expression of the *mini‐white* (*w*) marker (present in the pUAST vector) in their eyes. Successful integration of pUAST constructs at the intended genomic locations produces F_1_ flies with red‐ish eyes. Homozygotes were generated by intercrossing positive F_1_s and selecting F_2_s (males and virgin females) with darker red eyes. These were genotyped and intercrossed to establish the homozygous stock (Fig. S4).

### 2.12 Gal4‐mediated expression of UAS‐PxRyR

The UAS‐Gal4 system was used to drive the expression of PxRyR in *D. melanogaster* (following Brand and Perrimon, 1993^17^). Briefly, the expression strategy is as follows: the inserted pUAST‐PxRyR construct contains an Upstream Activation Sequence (UAS) under the control of the GAL4 transcription factor. Thus, the Drosophila line containing UAS‐PxRyR must be recombined with a Gal4‐containing line such that the GAL4 transcriptional activator is expressed and activates the UAS enhancer, as outlined in Fig. S5. The driving line employed was Bloomington Stock 67 480 [genotype y[1] w[*]; Mi{Trojan‐GAL4.0}RyR[MI08146‐TG4.0]/SM6a].^18^ Once it is integrated in the genome this trojan‐Gal4 co‐opts the transcription profile of the upstream regulatory region, whilst a poly‐adenylation sequence after the Gal4 halts transcription of the downstream region.^18^ Thus, knockout strains are generated that express GAL4 under the control of the regulatory elements of the knocked‐out genes. In this case, the Gal4 is inserted at base number 18 477 of the endogenous DmRyR genomic sequence, meaning that the Gal4 (and thus its UAS‐enhanced PxRyR sequence partner) is regulated by the same transcription factor machinery that regulates the endogenous DmRyR, whilst at the same time knocking out transcription of that endogenous DmRyR gene. Additionally, the *RyR* ^16^ allele (recombined into the injection strain; Fig. S3) is a deletion of the first intron of the DmRyR gene, thought to prevent functional channel formation.^15^ Thus, two different null‐RyR variants in combination are employed in order to knock out the endogenous RyR protein. The rationale behind the rescue strategy is that a Trojan‐GAL4‐RyR/RyR^16^ hemizygous strain is null, not viable and can survive only if a functional UAS‐RyR transgene is provided. Both Trojan‐GAL4‐RyR/Cy and RyR^16^/Cy also are homozygous lethal strains and only survive as heterozygotes because the balancer chromosome marked with Cy (curly wing phenotype) carries an intact DmRyR allele. Crossings between the RyR16.attP strain generated previously [y [1] M{vas‐ int.Dm}ZH‐2A w[*]; RyR [16]/CyO; M{3xP3‐RFP.attP}ZH‐86Fb] and the Trojan‐GAL4, as expected, generated only flies with Cy wings (Fig. S6). Non‐Cy Trojan‐GAL4‐RyR/RyR16 hemizygote flies were *RyR*‐null and not viable. By comparison, expression of UAS‐PxRyR via the RyR‐Gal4 driver was shown to successfully rescue the lethality caused by the lack of a functional DmRyR. This, however, was only achieved when crosses were kept at 17 °C and moved to 25 °C on the 9th day of development. Crosses kept at 25 °C did not generate any rescued individuals, possibly due to a leaky UAS promoter.^19^ Maintaining the fly crosses at a lower temperature until the 9th day of development helped to overcome lethality and allowed for the selection against the Cy phenotype, which is less pronounced at low temperatures. Non‐Cy, DmRyR null flies rescued from lethality by the expression of PxRyR genes were selected for bioassay and also used to generate stable fly strains (Fig. S7).

### 2.13 Confirming knock‐in and driving of PxRyR expression

Confirmation of successful knock‐in of PxRyR variants was via cDNA sequencing (Fig. S7, lower panel). Adult Drosophila RNA was extracted and used for cDNA synthesis using Superscript III (Life Technologies) and random hexamers (Life Technologies), according to the manufacturer's recommended protocol. The region of cDNA containing the mutation was amplified using primers PxRyR 11–13 (listed in Table S2). The sequenced region was divergent from that of *D. melanogaster* RyR and sequencing traces indicated that no amplification of *D. melanogaster RyR* cDNA had taken place.

### 2.14 Drosophila larval bioassays

Larvae for bioassay were reared under the following conditions, carefully controlling for larval density. Thirty adult virgins and 30 adult males of each strain were anaesthetized and placed into 8‐oz Drosophila stock bottles (Genesee Scientific), one bottle per strain, 17 days before bioassay. Bottles were incubated at 25 °C and adults allowed to lay eggs for 48 h, before being removed. After a further nine days at 25 °C, emerging adult males and virgin females were selected from each strain. Then 150 virgin females and 150 males of each genotype were placed into 8‐oz Drosophila stock bottles (two bottles per strain) which were incubated at 25 °C for 72 h. Stock solutions of CLR and FLB were made up at 4000 mg L^−1^ in 100% acetone. Stocks were diluted 1:50 to make up the first concentration (80 mg L^−1^) and diluted serially thereafter, at a ratio of 1:40, with all dilutions made in ddH_2_0 containing 2% acetone. For the noninsecticide control, ddH_2_0 containing 2% acetone was used. Narrow vials (Flystuff laboratory equipment, El Cajon, CA, USA) were pre‐prepared with 0.8 g dry fly diet (Nutri‐Fly Food, Genesee Scientific) per vial, with 3 mL of the relevant insecticide/control solution applied and incubated overnight for absorption of solution into the food and evaporation of acetone. The next day, 20 2nd instar (L_2_) larvae harvested from the Drosophila stock bottles were pipetted into each bioassay vial. In total six concentrations were tested and the bioassays were replicated thrice. The lethal concentrations necessary to kill 50% of the flies (LC_50_) after 72 h insecticide exposure were calculated by Probit analysis using genstat v18 (VSN International, Hemel Hempstead, UK).

### 2.15 Fecundity and fertility assays

One hundred newly emerged virgin females and 75 newly emerged males were placed into separate embryo collection cages, for each genotype (Genesee 59–101) with molasses agar plates (2% agar, 15% molasses, 0.8% propionic acid) supplemented with yeast paste to encourage egg‐laying. Flies were allowed to adapt in cages for a period of 48 h before beginning experimentation. After this point, eggs were extracted every 12 h by removal of the used egg‐plate from the cage and replacing with a new plate. Cages were maintained at 25 °C and egg extraction continued for three days. The used egg‐plate was labelled and incubated at 28 °C for 22 h to allow time for the fertilized eggs to hatch. Fecundity (number of eggs laid per cage per 24 h) and fertility (hatched eggs divided by the total number of eggs laid per cage per 24 h) were quantified for each plate of eggs. After quantification, egg‐plates were supplemented with additional yeast paste and returned to the incubator to develop into larvae. The experiment was replicated thrice.

### 2.16 Crawling assay

Around 20 L_2_ larvae were placed in the centre of a molasses agar dish (crawling arena) and subjected to 11 W halogen light at a distance of 15 cm from the dish edge. To stimulate crawling, a drop of yeast solution was placed in the middle of the well just before video recordings started. Negative phototaxis was recorded. Videos were recorded at 25 frames per s (fps) for ≈60 s, on a Sony HandyCam, 30 fps, mounted 50 cm above the dish. A virtual grid of 1 mm x 1 mm squares was placed over the recorded video using davinci resolve 15 (Blackmagic Design, Fremont, CA, USA) video editor. The paths of the larvae were tracked manually, and the number of squares passed through by each of the larvae was counted. Data were analyzed by ANOVA and least significant difference (LSD) in excel 2019 (Microsoft). The experiment was replicated twice.

### 2.17 Climbing assay

The climbing (negative geotaxis) assay was performed with the use of an automated fly‐climbing system.^20^ Briefly, the system employs a two‐storey acrylic tube rack capable of holding 20 standard Drosophila vials, each containing ten adult flies. The rack rests on a horizontal camshaft, with asymmetrical cams that cause the rack to rapidly rise and fall within a 4 mm travel as the shaft rotates, resulting in a violent and consistent shaking of the vials. The rack is marked with a horizontal line, at a height of 6 cm from the base of each vial, in order to assess fly‐climbing ability. Flies used for the assay were kept on standard fly food at 25 °C and transferred (by tapping, without the use of CO_2_) to empty vials and loaded into the automated climbing system. The climbing assay was carried out at 20 °C and involved cycles of: 5 s of vial shaking; 8 s for climbing; image capture; 45 s of resting, which were repeated 13 times during a single experiment. No data were collected during the first and third repeat to allow for habituation before data collection. Images were captured (13 s after the start) with a Canon EFS digital camera with an 18–55‐mm lens, positioned on a tripod at the same height as the centre of the climbing system. Captured images were scored manually to determine the number of flies above the 6 cm line in each vial and the score for each vial averaged over the ten data points. Data were analyzed by ANOVA and LSD in excel 2019 (Microsoft).

## 3 RESULTS

### 3.1 Generation of novel PxRyR constructs

**Figure 1:**
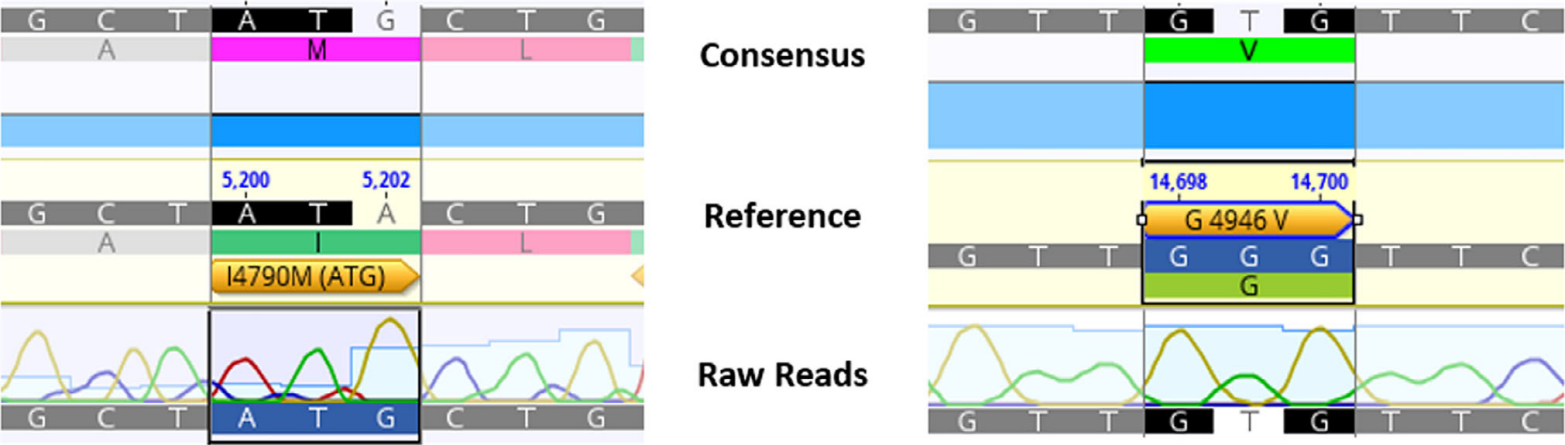
Sequence comparison of nucleic and amino acid positions for each novel PxRyR construct. ‘Consensus’ displays the successfully implemented alteration, whilst ‘Reference’ bears the original WT genotype. ‘Raw’ sequence data are also provided.

Two novel PxRyR constructs were created, introducing the relevant point mutations G/V and I/M at positions G4946 and I4790, respectively (Fig. 1). The properties of these *RyR* variants, when expressed in Sf9 cell lines, were compared against two previously created constructs, WT‐PxRyR and G4946E‐PxRyR.^13^

### 3.2 Functional expression of modified PxRyR constructs

**Figure 2:**
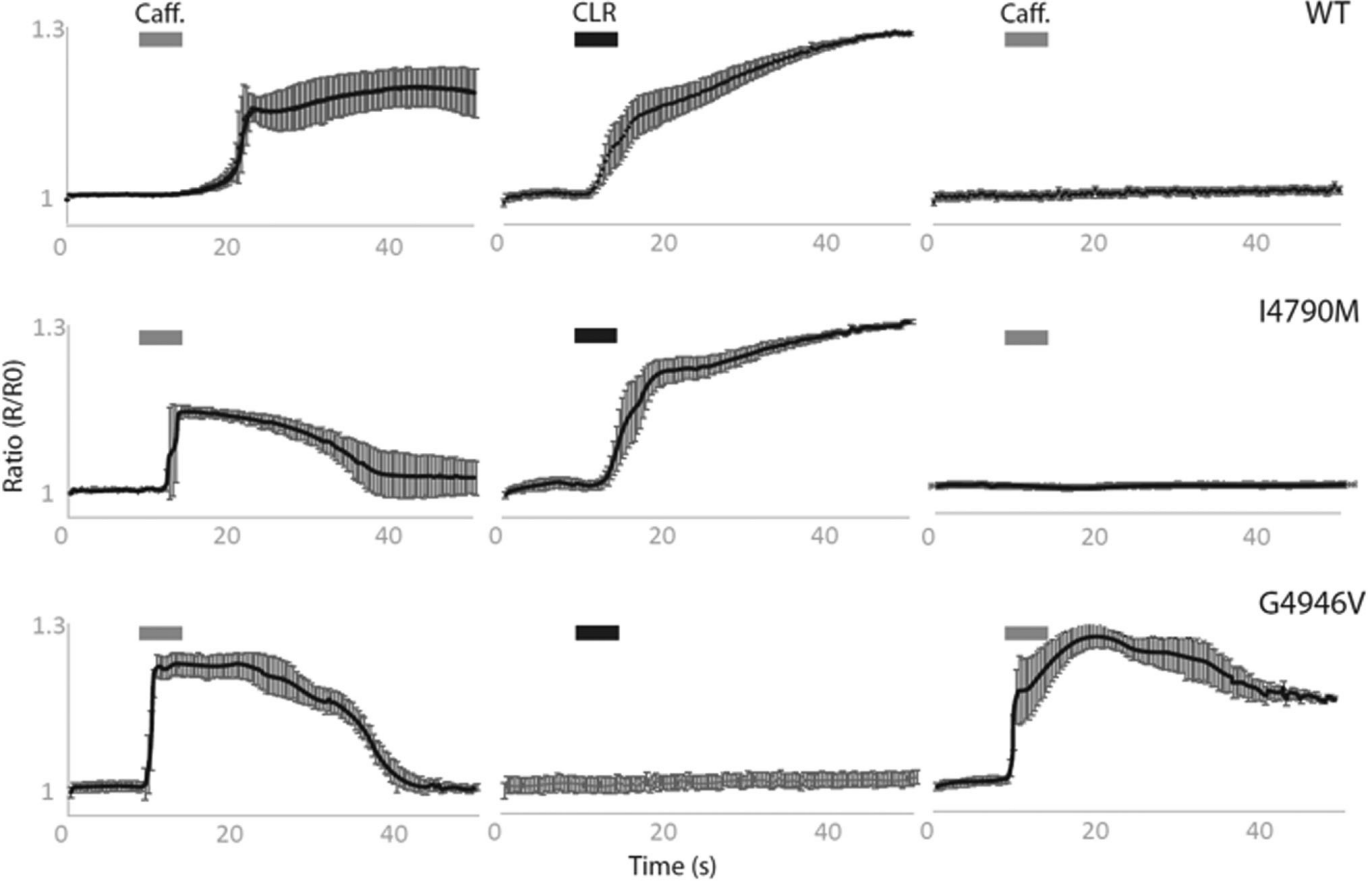
Ca^2+^‐release in Sf9 cells expressing recombinant PxRyR in response to the addition of 10 mmol L^–1^ caffeine (Caff.) and 5 μmol L^–1^ chlorantraniliprole (CLR). *R* represents the fluorescence ratio value recorded for individual time points and *R*_0_ the average fluorescence ratio calculated over the first 5 s before addition of the agonist. Error bars represent the SEM of cell response (*n* > 6).

Addition of 10 mmol L^–1^ caffeine to Sf9 cells expressing WT and modified PxRyR showed that all PxRyR variants form functional channels (Fig. 2). Subsequent exposure of the same cells to 5 μmol L^–1^ CLR revealed that CLR elicited Ca^2+^ release in WT‐ and I4790M‐PxRyR expressing cells but not in those cells expressing G4946V‐PxRyR. Subsequent addition of caffeine failed to provoke Ca^2+^ release in WT‐ and I4970M‐PxRyR previously activated by the diamide, but did elicit Ca^2+^ release in G4946V‐PxRyR cells. This effect was comparable to the response seen in G4946E‐PxRyR cells exposed to flubendiamide agonist reported previously.^13^ The postinsecticide caffeine triggered Ca^2+^ release determined in cells expressing G4946V (Fig. 2) and G4946E^13^ suggests that a mutation at this locus decreases the binding affinity for diamide insecticides. Mutation at I4790 does not mediate this same effect.

### 3.3 Ca^2+^ handling properties of WT and modified constructs

**Figure 3:**
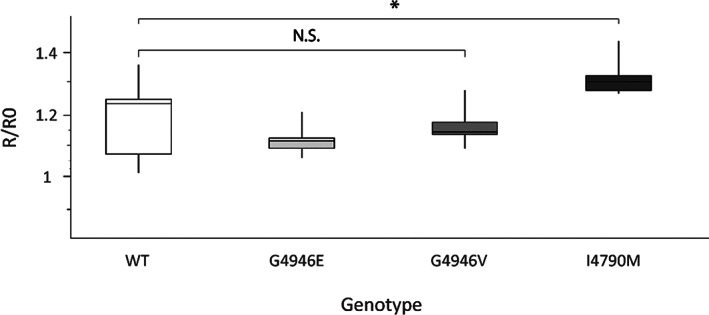
Comparative caffeine response between WT and modified PxRyR constructs. Boxplot of the comparative responses between PxRyR constructs. *R*/*R*_0_ is the relative increase in fluorescence in response to agonist application (maximum amplitude of each cell), normalized to pre‐agonist fluorescence. I4790M response to caffeine was found to be significantly higher than that of other genotypes (*n* = 9; *P* < 0.05).

Temporal and amplitude properties of caffeine evoked Ca^2+^ transients were characterized in Sf9 cells expressing WT and modified PxRyRs. In Sf9 cells carrying the G4946E‐PxRyR, basal cytoplasmic Ca^2+^, measured using ratiometric Fura‐2 Ca^2+^‐sensitive fluorescence, was significantly different when compared with WT PxRyR (340/380 ratio of 1.80 ± 0.32 *versus* 1.00 ± 0.38, respectively; *P* < 0.001, *n* = 11). Cytoplasmic Ca^2+^ levels in cells expressing PxRyR‐I4790M (1.51 ± 0.74) and G4946V (1.15 ± 0.21) were no different from WT PxRyR cells. Although several mechanisms, including trans‐ER and surface membrane Ca^2+^ fluxes, mitochondrial Ca^2+^ import and cytoplasmic Ca^2+^ buffering, contribute to Ca^2+^ homeostasis in Sf9 cells, these data are consistent with the G4946E mutation, but not the I4790M or G4946V mutations, producing gain‐of‐function PxRyR channels. The response of the WT and modified PxRyR to a single (10 mmol L^–1^) concentration of caffeine, which elicits Ca^2+^ release within the linear portion of the WT‐PxRyR caffeine‐response curve, indicated that neither G4946E‐PxRyR nor G4946V‐PxRyR produce a caffeine‐stimulated peak significantly different in amplitude to that of the WT‐PxRyR construct (*P* > 0.05; Fig. 3). However, the I4790M‐PxRyR produced a significantly higher peak (average R/RO: 1.31; *n* = 9; *P* < 0.05).

### 3.4 Impact of I4790M and G4946V mutations on PxRyR stimulation by diamides

**Figure 4:**
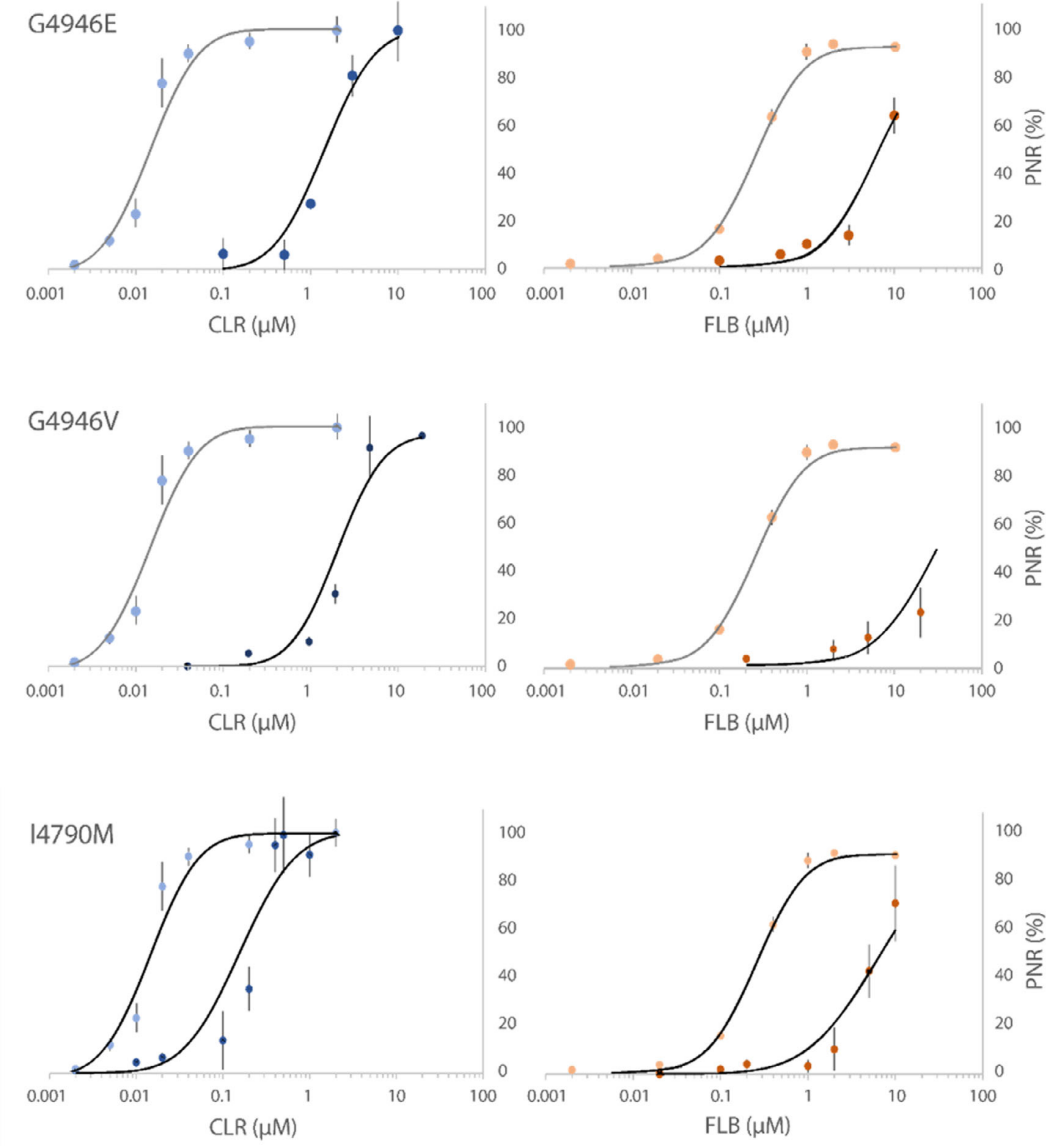
Concentration–response relationship of modified‐PxRyRs (dark fill) to CLR (blue) and FLB (orange), with WT‐PxRyR (blue & orange light fill) response included for comparison. The proportional normalized response (PNR) was calculated as described in Methods. Data points are presented as the mean ± SEM. For each concentration point, *n* = 5–8 clusters of cells, where a cluster contains between five and 32 caffeine‐responsive cells.

By successive application of caffeine and diamide, the recombinant PxRyR constructs were characterized in terms of their responsiveness to increasing diamide concentrations (Fig. 4; Table S3). The results obtained for the G4946E substitution corroborate previous work that reported a reduced efficacy of diamide interaction with the G4946E variant. The recorded resistance ratios (RRs) of 104‐fold for CLR was in line with the 218‐fold resistance reported by Troczka *et al*.^13^ for clonal Sf9 cells stably expressing this variant. As found previously,^13^ poor FLB‐solubility (despite using the more water‐soluble sulfoxide FLB analog) precluded exact *quantitative assessment of FLB RR in all of the PxRyR variants tested here. The G4946V* substitution likewise mediated substantial resistance to CLR. A calculated RR of 146‐fold to CLR indicates a somewhat more potent resistance effect than seen for G4946E. However, caution should be exercised in comparing the resistance profiles of the two constructs given that a detailed analysis of cell physiology was not made in this study. I4790M also conferred a degree of resistance to diamide insecticides. For CLR, the calculated RR was moderate, at just ten‐fold, whereas a higher resistance was apparently conferred to FLB, estimated as >24‐fold, which is comparable to an estimated RR for G4946E of >23‐fold.

### 3.5 *Drosophila melanogaster* I4790M studies

In order to corroborate the findings from the Sf9 assays above it was important to show that reductions in diamide efficacy (Fig. 4) conferred by I4790M *in vitro* were recapitulated *in vivo*. Several other authors previously have succeeded in demonstrating that a resistance phenotype is conferred by this *RyR* alteration *in vivo*. Notably, Douris *et al*.,^21^ using reverse genetic studies in *D. melanogaster*, substituted the naturally occurring methionine at position 4790 of the Drosophila *RyR* with isoleucine (M4790I), which conferred a 7.5‐fold increase in CLR efficacy and a 15‐fold increase in FLB efficacy. Meanwhile, other studies have focussed on altering the pest of interest directly. A backcrossing experiment in *S. exigua* found that introgression of the I4790M mutation into a WT line caused a ≈20‐fold resistance to both CLR and FLB.^8^ In the present study, a novel approach was taken, whereby the entire lepidopteran coding sequence of a target‐site resistant (I4790M) and nonresistant (WT) *RyR* isoform from *P. xylostella* was inserted into *D. melanogaster*. This is the first report of an entire *RyR* sequence being cloned and inserted into a different species.

**Figure 5:**
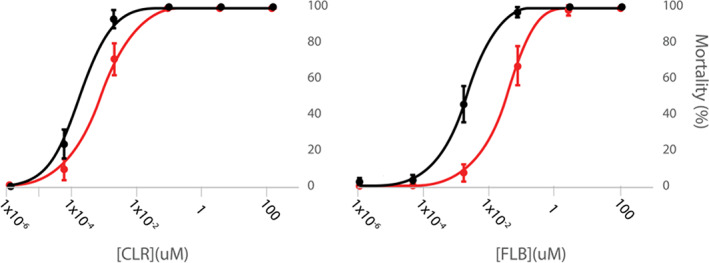
Nonlinear dose–response curves for two diamide insecticides. Mean mortality of fruit fly larvae homozygous for WT‐PxRyR (black) or I4790M‐PxRyR (red) after 72 h exposure to increasing doses of CLR or FLB. Error bars indicate 95% CI.

A toxicological impact assessment of the diamides CLR and FLB was carried out on the PxRyR WT and I4790M fly lines by conducting larval bioassays. It was seen that flies homozygous for I4790M‐PxRyR showed minor resistance to CLR (Fig. 5; Table S4). The LC_50_‐value of CLR against I4790M‐PxRyR flies was 4.44‐fold higher than that necessary to kill 50% of WT‐PxRyR flies (RR 4.44). By contrast, I4790M‐PxRyR flies exhibited a markedly higher level of resistance to FLB, with an LC_50_ based RR of 22‐fold. The fly lines created corroborate the impact of the I4790M mutation upon diamide efficacy.

### 3.6 Fitness costs associated with I4790M

Having determined the impact of the I4790M mutation on diamide efficacy in Drosophila larvae, we sought to understand whether the altered response to diamide was coupled to any other physiological alterations. In the context of field control, decreased mortality during insecticide exposure, as conferred by the I4790M or G4946E/V mutations, converted into a selective advantage for the individual compared to WT individuals. However, previous studies have indicated that fitness benefits, in the presence of insecticide treatment, are expected to be balanced against fitness costs in the absence of insecticide treatment.^22^

Fecundity/fertility and developmental success are two key components of lifetime reproductive success. These two parameters were studied for each Drosophila PxRyR genotype (WT *versus* I4970M) under noncompetitive conditions (i.e. genotype alone in a cage). Because the fecundity/fertility is a noncompetitive assay, it clearly does not consider the potential impact of the mutation upon competition for limited resources including adult nutrition, competitive mating success, competitive egg laying and larval nutrition. In order to attempt to account for the impact of the mutations upon competition, we studied two independent indicators of vigour: speed of larval movement and adult climbing ability. It can be presumed that success in most aspects of competition (as listed above) is mediated by the ability to move, be that moving toward a food source, toward a potential mating partner, toward an optimal egg laying location, or away from predators.

#### 3.6.1 Fecundity and fertility

**Figure 6:**
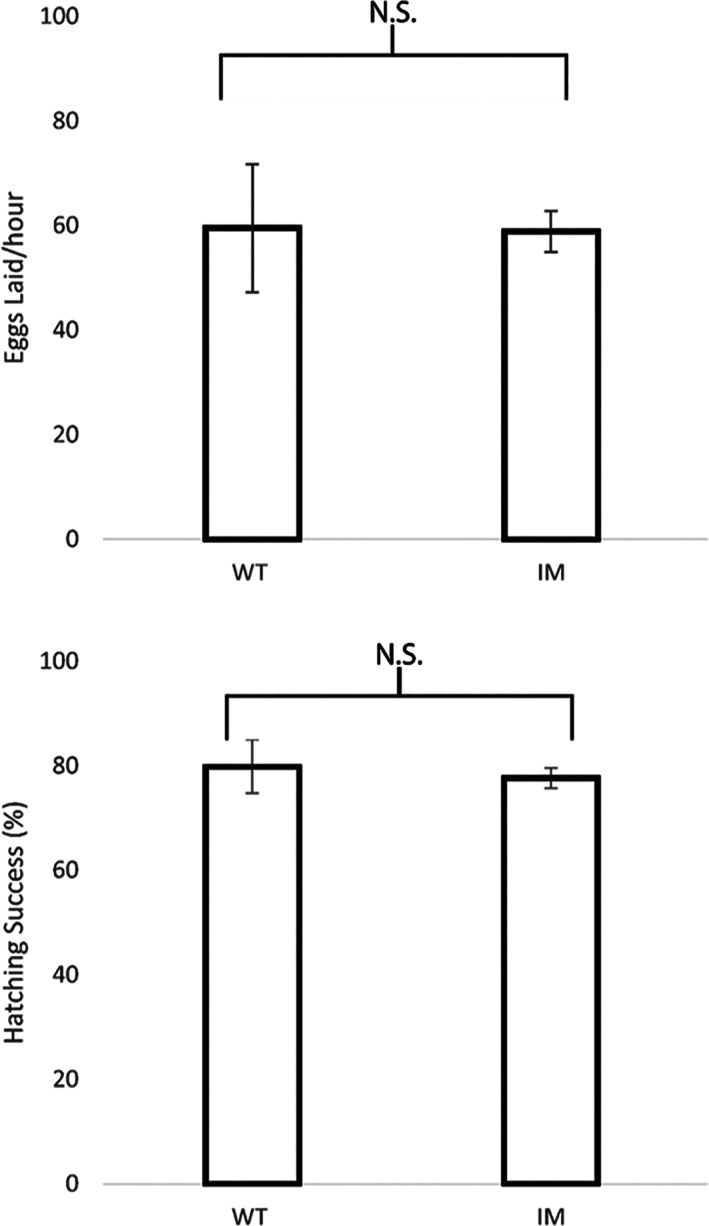
I4790M diamide‐resistant *D. melanogaster* do not differ in fecundity or fertility compared to their WT counterpart. Bar graphs of total fecundity (top panel) and proportion of eggs hatching within 24 h (lower panel) at 25 °C. *n* = 3 plates, from cages of 175 flies. Error bars indicate SD.

No differences were found in indicators of reproductive success between the fly lines (Fig. 6). Cages of WT flies laid a median 60 (±10) eggs h^–1^, compared to I4790M 59 (±3) eggs h^–1^ (ANOVA, *F*_crit_ = 9.1, *F* = 0.51). Fertility varied between 75% and 85% successfully hatching eggs across both lines. There was no significant difference in the proportion of larvae successfully pupating [WT 72 (±13)%; I4790M 65 (±14)%) (ANOVA, *F*_crit_ = 5.4, *F* = 2.6)] nor in the proportion of pupae successfully eclosing [WT 69 (±19)%; I4790M 77 (±11)%) (ANOVA, *F*_crit_ = 5.4, *F* = 1.9)].

#### 3.6.2 Crawling speed and climbing ability

Indicators of vigour were found to differ significantly between the fly lines, with the I4790M line showing signs of reduced movement relative to flies expressing WT‐PxRyR.

**Figure 7:**
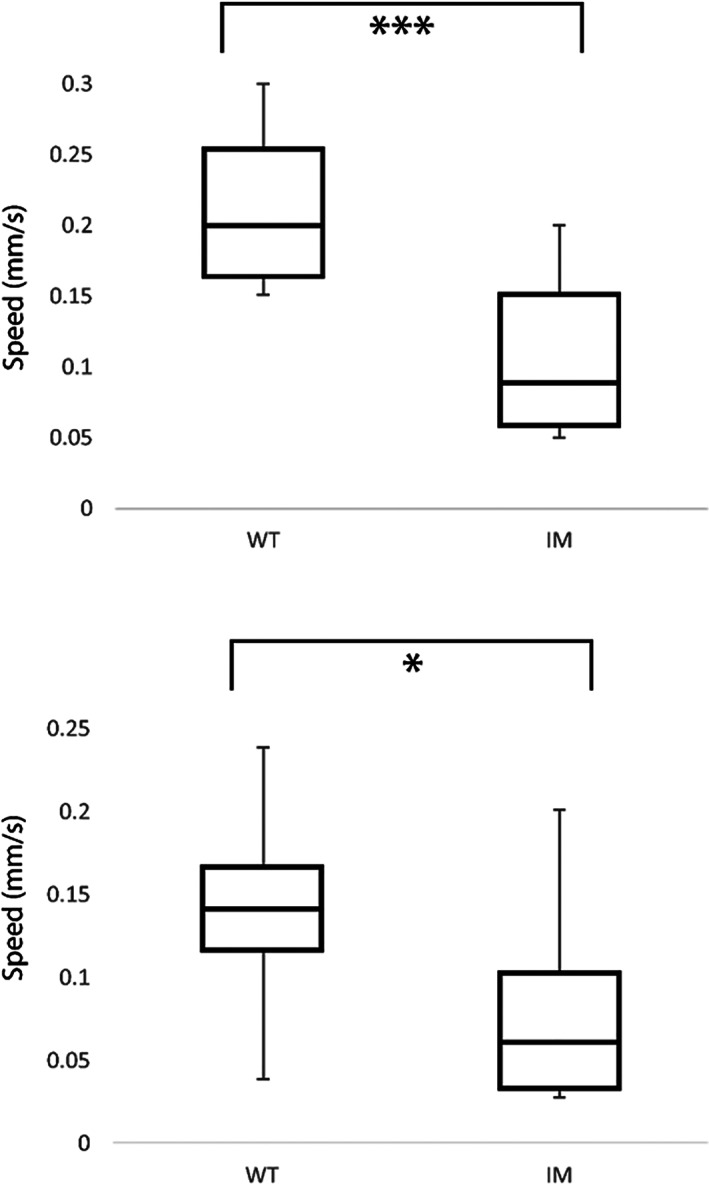
I4790M diamide‐resistant *D. melanogaster* larvae crawl significantly more slowly than their WT counterparts. Boxplots indicating maximum (upper panel) and average (lower panel) crawling speed. ***, *P* < 0.001; *, *P* < 0.05 determined by ANOVA and LSD. *n* = 20 larvae per genotype; the experiment was replicated (*n* = 2), and similar results were obtained.

L_3_ larvae have well‐developed crawling musculature and naturally exhibit migratory behaviour, searching for a suitable location for pupation. Accordingly, larvae were placed in an environment of high light intensity, zero food availability and no shelter, encouraging migratory behaviour, which was measured for 3 min. The maximum speed achieved by each larva during the time window (averaged over a 15‐s period) is plotted in Fig. 7 (upper panel). WT larvae crawled at a velocity of 0.21 (±0.05) mm s^−1^, more than double the maximum velocity of 0.10 (±0.06) mm s^−1^ of I4790M larvae. The average speed during the 3‐min period is plotted in Fig. 7 (lower panel). In this measure, too, the difference between lines is significant, with WT larvae averaging 0.14 (±0.06) mm s^−1^, more than double the distance of I4790M larvae at 0.06 (±0.05) mm s^−1^.

**Figure 8:**
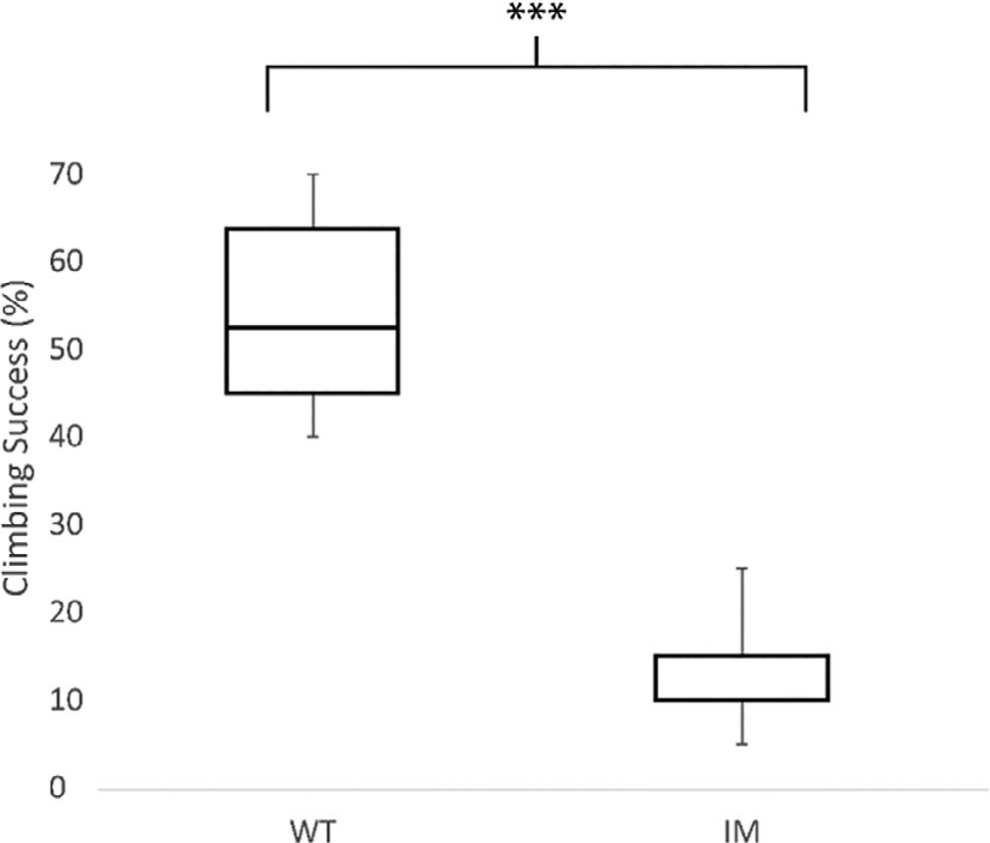
Boxplot of climbing success between PxRyR‐expressing *D. melanogaster* lines. The I4790M diamide‐resistant genotype climb more slowly than their WT counterpart. ***, *P* < 0.001 as determined by ANOVA and LSD. *n* = 40 flies per genotype; the experiment was replicated, and similar results were obtained.

Adult cohorts from each genotype were raised in identical conditions used for the fertility/fecundity assays. Adults were tapped into vials, shaken and knocked to the base of the vial using a Hillary climber apparatus, before being allowed to climb the vial walls. The proportion successfully climbing above a 6 cm threshold after 8 s was recorded (Fig. 8). WT adults successfully climbed above the threshold 40–70% of the time (median 53%), whilst I4790M adults managed the same feat less than half as frequently (median 12%).

In summary, the results indicate that whilst the PxRyR alteration appears to bear no fitness costs in terms of reproductive capacity in a noncompetitive environment, the I4790M substitution has potentially major effects on flies' ability to move. In the context of a competitive ecosystem, I4790M diamide resistant individuals are likely to suffer a considerable fitness cost.

## 4 DISCUSSION

It has been shown, over the past decade, that the spread of diamide insecticide resistance represents (in many cases) the spread of allelic variants of the *RyR* gene that exhibit reduced diamide interactions. There are currently three variants of particular relevance to the spread of resistance within lepidopteran pest populations.

### 4.1 G4946E – the root of resistance

Diamide resistance associated with the PxRyR substitution G4946E in the field has been shown to vary from ≈2000‐fold for FLB in China, to ≈10 000‐fold in the Philippines,^23^, ^24^ whilst resistance to CLR appears to be ≈775‐fold.^24^ Over the past decade, the role of G4946E in diamide resistance has been characterized extensively *in vitro*. Sf9 cells expressing an insecticide‐susceptible WT‐PxRyR channel exhibited elevation in cytoplasmic Ca^2+^ levels in response to 100 nM FLB application, whilst those expressing the G4946E variant were refractory to such effects up to (and most likely beyond) the limit of solubility of the compound.^13^ For CLR, a more complete concentration–response profile was achieved for both the WT‐PxRyR and G4946E‐PxRyR constructs, with the EC_50_ increasing from ≈0.017 μm to ≈3.7 μm, implying a RR of 218‐fold (roughly comparable to the 104‐fold resistance recorded in the present study). Likewise, native membrane preparations containing PxRyR from a resistant moth strain exhibited 450‐fold (FLB) and 159‐fold (CLR) reduced binding when compared to membrane preparations from a susceptible strain.^24^

### 4.2 G4946V – a novel G4946 resistance variant in *T. absoluta*

Reports of a tomato leafminer (*T. absoluta*) outbreak in Europe were accompanied by sequencing of a novel diamide resistance‐associated variant, G4946V in *T. absoluta RyR*.^5^ Populations of *T. absoluta* with diamide resistance ratios for CLR of ≤2700‐fold or >3200‐fold subsequently were recorded in Italy and Greece (respectively).^11^ It was demonstrated that *T. absoluta* membranes harbouring G4946E/V‐RyRs had FLB binding reduced by >300‐fold.^5^ This novel G4946V mutation characterized in the resistant *T. absoluta* populations has not yet been identified in other species. It was necessary therefore to experimentally validate the role of the mutation (in isolation) in the observed resistance episodes. The mutation also is interesting in the context of achieving a better understanding of the nature of G4946E‐mediated resistance. The valine (V) substitution in the *T. absoluta* populations has no overall charge associated with it, compared to the strong negatively charged glutamic acid (E) substitution. As both appear to cause an approximately equal level of resistance in Sf9 cell studies (100‐ to 150 ‐fold), this might indicate that the associated decrease in diamide efficacy predominantly is due to binding site obstruction rather than changes in chemical interaction. These resistance values recorded correspond favourably with those found by Douris *et al*.,^21^ who used CRISPR/Cas9 to generate modified *D. melanogaster* flies bearing the G4946V mutation which exhibited high resistance ratios to flubendiamide (91.3‐fold) and chlorantraniliprole (194.7‐fold).

### 4.3 I4790M – an alternative locus of resistance in lepidopteran pests

Following on from the characterization of the G4946E variant in *P. xylostella*,^1^ three novel mutations E1338D, Q4594L and I4790M were reported in Yunnan province, China, in *P. xylostella* individuals displaying a 2128‐fold resistance to CLR.^6^ However, it soon became apparent that I4790M was of much greater relevance than the other two mutations, emerging autonomously in various lepidopteran species and isolated populations when subjected to diamide selection. In the field its presence is associated with ≈150‐fold resistance to CLR.^8^ However, near‐isogenic I4790M (*P. xylostella* numbering) laboratory‐generated strains of *S. exigua* showed only moderate levels of resistance to CLR (21‐fold) and FLB (22‐fold), suggesting that the I4790M mutation confers moderate levels of resistance to diamide insecticides. This was confirmed in the present study where the I4790M change was found to mediate a five‐ to ten‐fold reduction in CLR efficacy, and resistance to FLB was estimated as being between 20‐ and 35‐fold [as determined by both *in vitro* (Sf9) and *in vivo* (*D. melanogaster*) expression studies]. Another study that compared CRISPR/Cas9 modified *D. melanogaster* carrying an isoleucine substitution in the native Drosophila *RyR* with flies naturally WT for the I4790M mutation, showed that the latter likewise were moderately resistant to CLR (7.5‐fold) and flubendiamide (15.3‐fold).^21^ A more recent study, using a CRISPR/Cas9 knock‐in homozygous strain (I4790M‐KI) of *P. xylostella*,^25^ found that the manipulated I4790M‐KI strain exhibited moderate resistance to CLR (6.0‐fold) and a slightly more elevated resistance to FLB (40.5‐fold). The collective observations from these independent studies suggest that the I4790M mutation confers a greater degree of resistance to FLB than CLR and provide support for the premise that the I4790M locus at least partially accounts for diamide insensitivity in insect orders outside the lepidoptera. However, it is important to note that the diamide resistance conferred by I4790M in these studies is much less severe than the RRs calculated previously for lepidoptera *versus* other insect orders, which can reach a magnitude of 10 000‐ or 100 000‐fold difference,^26^ even accounting for the observation that field‐study RRs tend to be much higher than laboratory‐study RRs. If the I4790M is a selectivity switch, it must be one of several such amino acids on the *RyR* channel which act in concert to confer diamide selectivity. Recently, a second substitution I4790K has been described in *P. xylostella* populations in Japan^27^ and Australia,^28^ implying that this is an important locus that is continually being selected in response to selection pressure imposed by exposure to diamide insecticides. Resistance levels conferred by the I4790K mutation have been shown to be extremely high (I4790K > G4946E > I4790M).^29^

**Figure 9:**
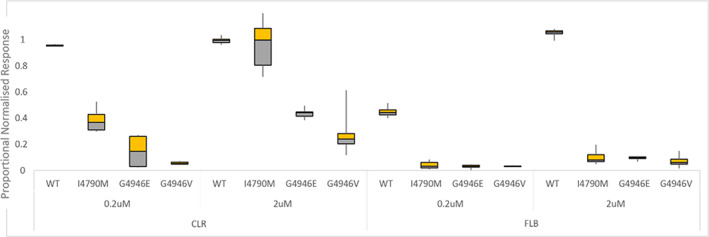
Boxplot comparison of all resistant PxRyR variant responses to diamides CLR and FLB. Error bars represent SEM.

To conclude, the results that we obtained show that the substitutions G4946E/V and I4790M on PxRyR cause a significant reduction to the diamide effect in PxRyR‐expressing Sf9 cell lines (summarized in Fig. 9). The benefit of *in vitro* studies such as these, where the comparison is made between PxRyR variants that differ by just a single amino acid alteration, is that it provides a tractable experimental background totally isolated from the compensatory mechanisms that might exist *in vivo*. Although recent reverse genetic *in vivo* transgenic studies^21^, ^25^ have provided us with insight into the impact of the I4790M RyR mutation on diamide resistance *in vivo*, this study further corroborates and extends previous findings using both *in vitro* and *in vivo* approaches.

### 4.4 I4790M‐mediated resistance may have a cost

The *D. melanogaster* lines generated in this study also were used to investigate the fitness costs associated with the I4790M mutation. An I4790M change introduced into the protein was found to significantly hinder larval crawling ability and significantly slow adult climbing speed (as a caveat, however, it should be noted that these data are collected in a comparison of *Drosophila* strains expressing variants of the *P. xylostella* RyR, rather than in the native moth). Significantly, Ca^2+^ release through RyR is the cardinal event in muscular contraction, and the results shown here clearly signpost that altered ‘gain‐of‐function’ Ca^2+^ handling mediated by the I4970M mutation (Fig. 3) is associated with a negative impact on locomotor activity (Figs 7 and 8). The data obtained would be consistent with the I4790M mutation establishing a cellular Ca^2+^ environment akin to that resulting from mutations in mammalian *RyR1* that are causative of the skeletal muscle pathologies malignant hyperthermia (MH) and central core disease (CCD).^30^ Indeed, two MH‐ and CCD‐linked mutations on *RyR1*, at Y4795 (CCD) and G4819 (MH/CCD), coincide with the positions of other reported diamide resistance mutations Y4922F and G4946E in lepidoptera,^15^, ^31^ and another mutation at R4563 (MH) has been strongly implicated in mammalian diamide insensitivity.^32^ The equivalent residue in lepidoptera (K4700, *P. xylostella* numbering) recently has been identified as the key residue for diamide interaction on the insect channel.^32^ There is evidence to suggest therefore that the diamide binding site in insects corresponds to a region linked to autosomal dominant skeletal myopathies in humans, and that the underlying genetic locus is a mutational hot spot across species.

No detrimental impact associated with I4790M was observed on fecundity and fertility, in line with recent studies that examined the biotic performances of diamide‐resistant *P. xylostella* laboratory‐introgressed^28^ and field‐collected strains.^33^, ^34^ The results published therein showed that the diamide‐resistant strains carrying G4946E or I4790K mutations exhibited hatchability, larval development and fecundity equivalent to those of diamide‐susceptible strains.

## 5 CONCLUSIONS

In lepidopteran field populations placed under increasing selection pressure, it is postulated that multiple target‐site resistance mechanisms can combine to aggravate the diamide resistance phenotype. The contributions of each individual mutation to resistance is now becoming clearer, as are subtle differences in the resistance levels conferred to anthranilic (CLR) *versus* phthalic acid (FLB) diamides by the different resistance loci.

## CONFLICT OF INTEREST

The authors declare no competing interests.

## Supporting information


**Supporting information may be found in the online version of this article**.

## Data Availability

The data that support the findings of this study are available from the corresponding author upon reasonable request.
