# Supplementary material for: Diamide insecticide resistance in transgenic Drosophila and Sf9‐cells expressing a full‐length diamondback moth ryanodine receptor carrying an I4790M mutation
Source: Pest Manag Sci. 2021 Dec 3;78(3):869–80. doi: 10.1002/ps.6730 (PMC9255861; doi:10.1002/ps.6730)
Supplement: Supplementary file 1 — Supporting information may be found in the online version of this article. [file PS-78-869-s001.docx]

**SUPPORTING INFORMATION**

**Supplementary Methods M1**

To facilitate fragment reassembly, a hybrid plasmid vector was used, composed of the multiple-cloning-site (MCS) from pcDNA3.1(-) (Invitrogen, Carlsbad, CA, USA), plus the addition of two extra restriction enzyme cut sites (HindIII and MluI cut sites) at the 5’ and 3’ ends of the MCS respectively, spliced into a pIZ/V5-His plasmid vector (Thermo-Fisher Scientific, Waltham, MA, USA) in place of its original MCS. The four fragments (F1 to F3 and a modified F4) to be re-incorporated into the linearised hybrid pIZ/V5-His plasmid vector where then re-assembled into a whole intact full-length cDNA corresponding to the PxRyR ORF by ligation in the presence of the cut hybrid pIZ/V5-His plasmid vector. The total DNA concentration in the ligation reaction was maintained at ~10ng/ul. The addition of HindIII and MluI cut sites at the 5’ and 3’ ends of the pcDNA3.1(-) MCS, allowed the amplified sequence reassembled in the hybrid vector to be easily transferred across into pIZ/V5-His.

**Supplementary Methods M2**

A data analysis pipeline consisting of the following steps was used to calculate the PNR to diamide insecticides for each responding cell.

Identify caffeine-responsive cells:

For each cell, the caffeine response was measured as follows:

- - Calculate ‘average fluorescence prior to application’: **R_0_**
  - Calculate ‘normalised fluorescence score at each timepoint’ for responding cells: **R/R_0_^response^**
  - Calculate and subtract ‘cellular background’: (**R/R_0_**)**^total^** = (R/R_0_)^response^ - (R/R_0_)^non-response^
  - Calculate ‘maximum fluorescence amplitude’: **∆(R/R_0_)[Caffeine]** = Max(R/R_0_^total^) - 1

Similarly, measure response to diamide as follows:

- - Calculate **R_0_**
  - Calculate **R/R_0_**
  - Calculate **∆R/R_0_ [Diamide]**

For each cell, divide **∆R/R_0_ [Diamide/Caffeine]** to get the PNR triggered by diamide

The above was repeated for all cells in the FOV, for the given dose, and the mean average PNR across all cells in the FOV calculated.

Note: In order to allow for comparison of I4790M responses against the other genotypes included in this study, caffeine responses for I4790M were adjusted downward by 31%. This adjustment was required due to the methods used to determine a diamide concentration-response, which involves calculating the Proportional Normalised Response (PNR) of a cell to first caffeine and then diamide.

**Supplementary Methods M3**

Newly laid embryos were rinsed off the egg-laying plate into a mesh basket and washed with water. Embryos were then transferred to a glass cover slip and aligned, using a fine sable paintbrush, with the dorsal side face up and the posterior end of the embryo ~2 mm from the edge of the coverslip. Aligned embryos were dried until the aqueous meniscus between eggs was on the verge of disappearing (30s-5min). To halt the drying process halocarbon oil 27 (Sigma-Aldrich) was applied sparingly to the embryos. Prior to injection, embryos were incubated for a further 5 minutes to allow the halocarbon oil to penetrate between the chorion and vitelline membrane. pUAST-PxRyR constructs were microinjected into the non-decorionated syncytial blastoderm embryos using an inverted microscope (Eclipse TieU, Nikon, Japan) equipped with a 10x/0.25 (magnification / aperture) lens, 10x/22 eyepiece and ﬂuorescence illumination. The injection solution contained 150ng/µl UAS-PxRyR plasmid and 100ppm fluorescent dye (fluorescin isothiocyanate dextran, Sigma- Aldrich) in injection buffer (0.5x phosphate buffer pH 6.8 (0.05 mM sodium phosphate, 2.5 mM KCl)). Solutions were delivered into the embryo by a FemtoJet express microinjector (Eppendorf, Hamburg, Germany) controlled by a motorised TransferMan NK2 micromanipulator (Eppendorf, Hamburg, Germany). Injection needles were prepared from quartz capillaries (WPI, D=1mm, L=100mm) using a P-2000 micropipette puller (Sutter Instrument Co, Novato, USA). The injection needle was back filled with 0.5μl of injection solution and aligned to the embryo posterior.

**Table S1.** Primers used to generate amino acid substitutions in the *P. xylostella* RyR.

|  | | |
| --- | --- | --- |
| Mutation | **Oligonucleotide 1** | **Oligonucleotide 2** |
| G4946V | GGACGTGGCTGTTGTGTTCAAGACGTTGAGGAC | CTCAACGTCTTGAACACAACAGCCACGTCCAACAG |
| I4970M | GTATCGCTGGCTATGCTGATCGGGTACTACC | TAGTACCCGATCAGCATAGCCAGCGATACTATAGAG |

**Table S2.** PxRyR sequencing primers

| PxRyR F11 | GACTCACGCTTCCGGTTTAATGG |
| --- | --- |
| PxRyR R11 | CTGCGCCTCTATCCTCTCTTGAG |
| PxRyR F12 | TGGTGAACAAGCCAAGAAGCAAG |
| PxRyR R12 | AAGAACCCTCTGCCCTCATCTTC |
| PxRyR F13 | TCAAGCGTGAGAAGGAGATAGCC |
| PxRyR R13 | CCTAAGTCTACTCTCCCATGGCG |

**Table S3.** Concentration-Response data for FLB and CLR applied to Sf9 cells expressing modified PxRyRs

| Compound | Genotype | Slope | LC50 (μM) | RR |
| --- | --- | --- | --- | --- |
| FLB | WT | 11.2 | 0.28 |  |
|  | G4946E | 6.3 | >6.5 | >23  *†*  *†*  *†* |
|  | G4946V | 6.5 | >30 | >100 |
|  | I4790M | 5.1 | >6.8  *†*  *†* | >24 |
| CLR | WT | 11 | 0.014 |  |
|  | G4946E | 10.8 | 1.46 | 104.3 |
|  | G4946V | 7.1 | 2.05 | 146.4 |
|  | I4790M | 7.33 | 0.145 | 10.4 |

*‡ Resistance Ratio (Genotype LC50 divided by WT LC50)*

*‡*

*†*

*† It was not possible to complete this concentration-response due to reaching the solubility limit of the FLB compound*

**Table S4.** Concentration-Response data for FLB and CLR tested against fruit fly larvae homozygous for WT-PxRyR or I4790M-PxRyR after 72hr exposure

*‡*

| Compound | Genotype | Slope | LC50 (μM) | RR |
| --- | --- | --- | --- | --- |
| FLB | WT | 5 | 0.002 |  |
|  | IM | 4.7 | 0.044 | 22 |
| CLR | WT | 3.9 | 0.00018 |  |
|  | IM | 3.8 | 0.0008 | 4.44 |

*‡ Resistance Ratio (Genotype LC50 divided by WT LC50)*

**
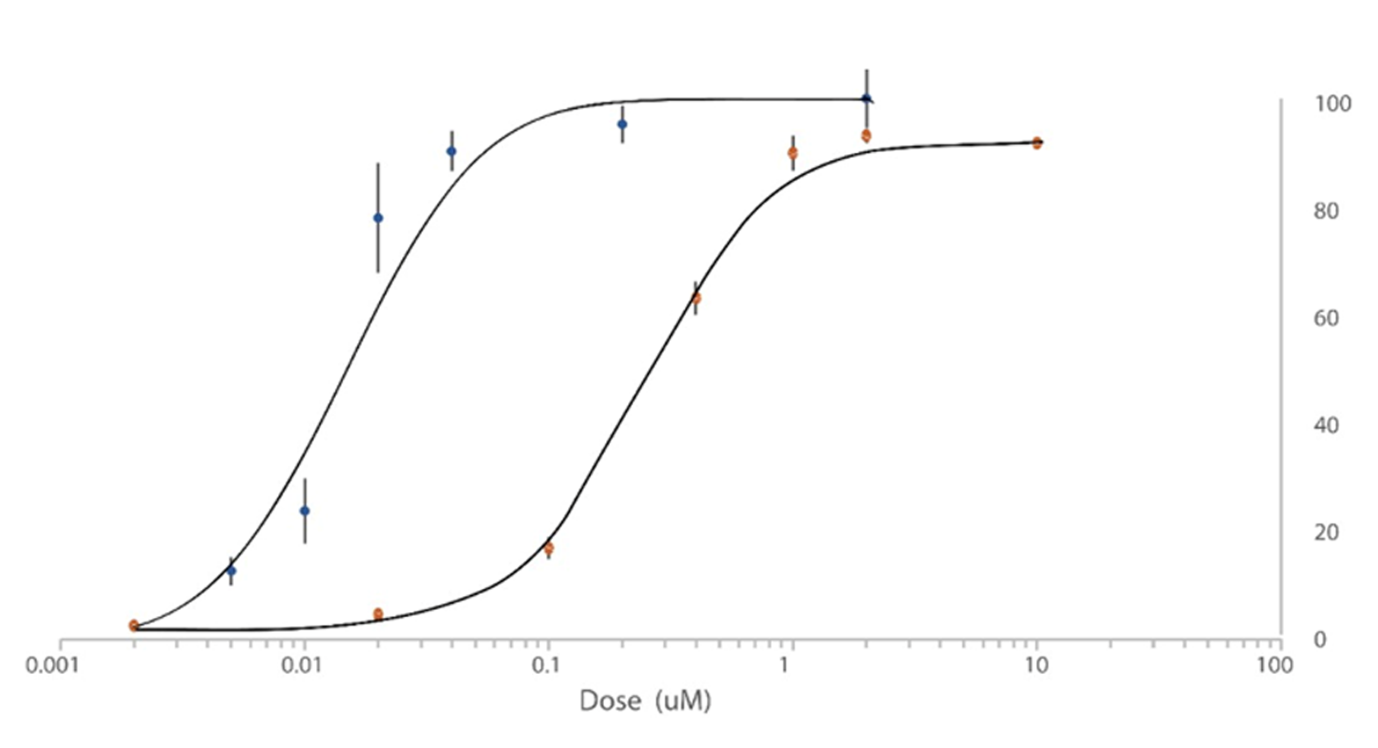
**

**Figure S1.** Concentration-response curves showing the normalised response of WT-PxRyR expressing cells to stimulation by CLR (blue) and FLB (orange).


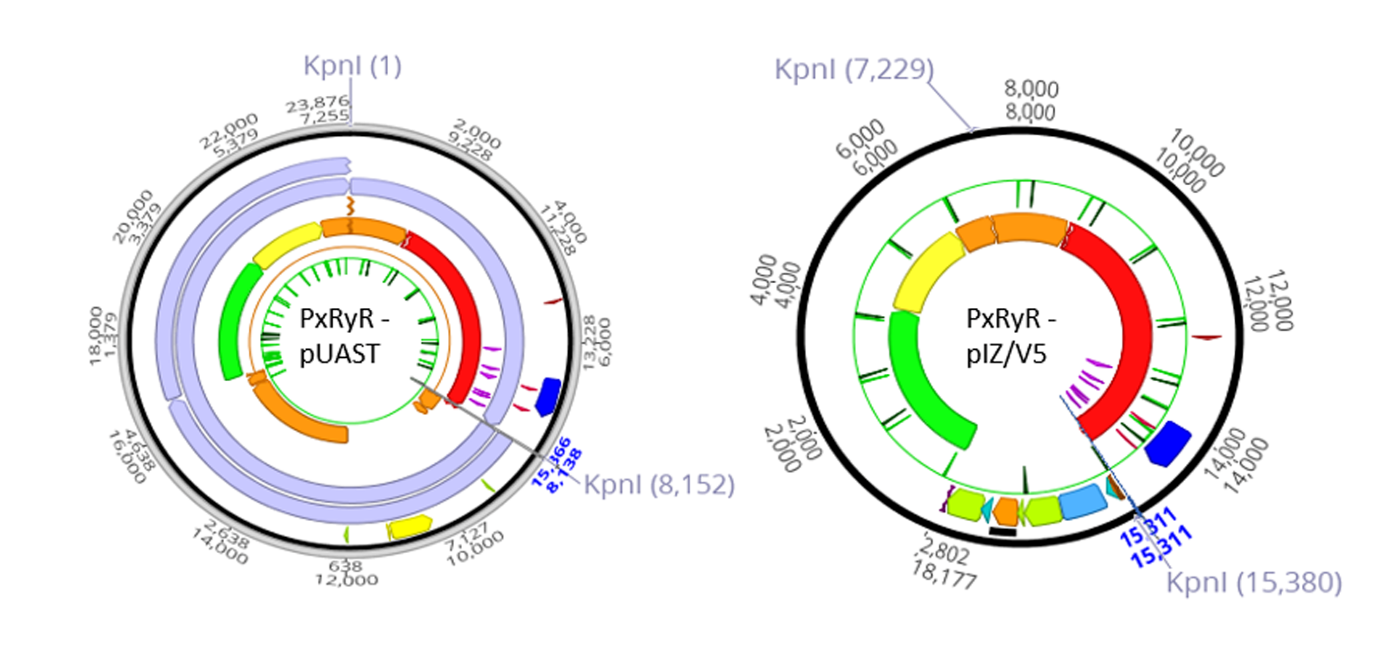


**Figure S2.** Transfer of PxRyR C-terminal fragments between constructs of different vectors via KpnI digestion.

**
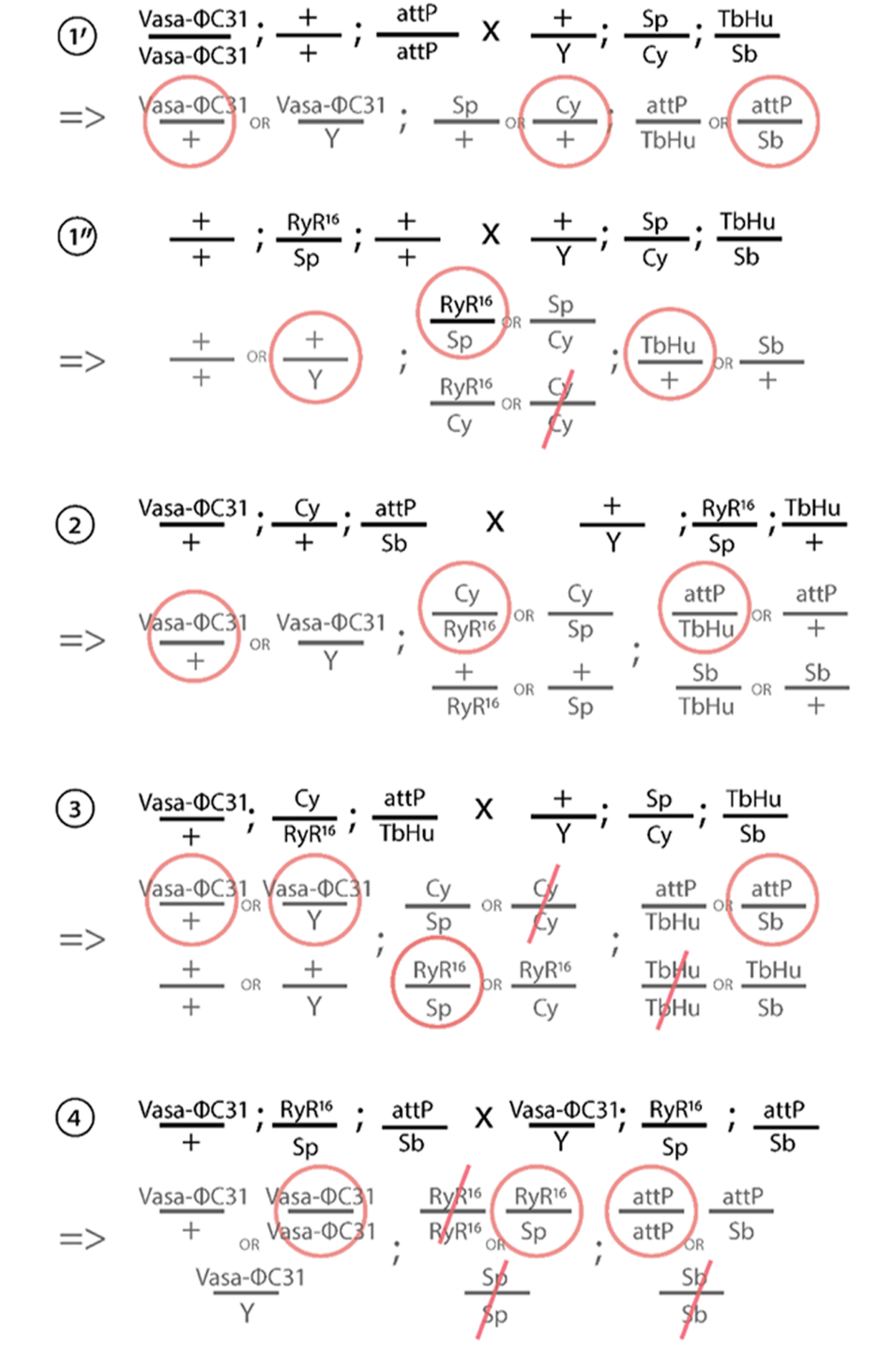
**

**Figure S3.** Iterative crossing with a double-marker line was used to combine two Bloomington stock lines to create an injection line that expresses ϕC31 by the *vasa* germline promoter (Chromosome 1), the RyR16 knockout (Chromosome 2) as well as the attP sequence for UAS-mediated genomic integration (Chromosome 3). Genotypes selected for further crosses are circled in red. A red strike through indicates a non-viable genotype.


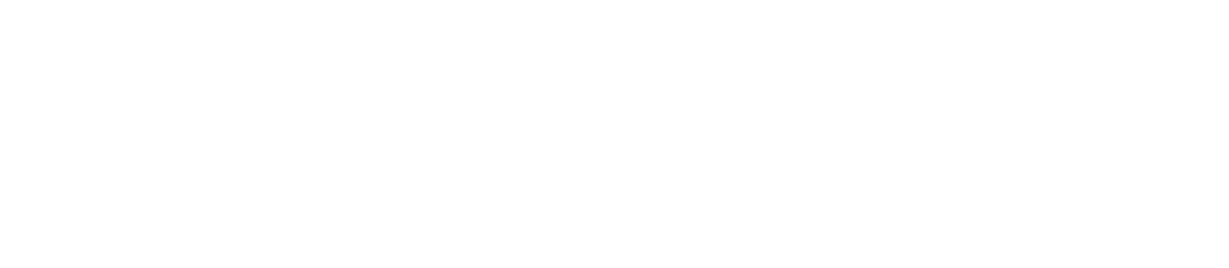


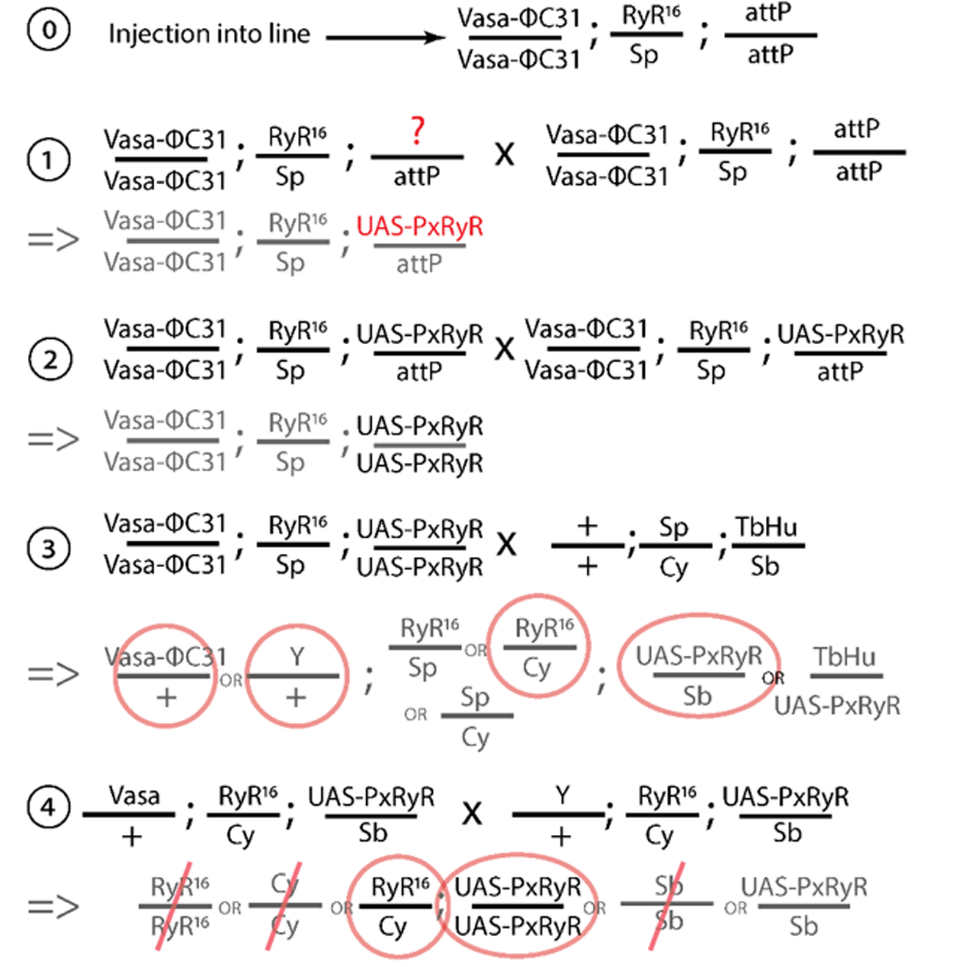


**Figure S4.** RyR16.attp line was injected with UAS-PxRyR plasmid DNA to achieve integration of the construct. Integrated F0 lines were back crossed to the RyR16.attp line to form heterozygous PxRyR (F1). Chromosomal swapping via the double-marker strain was used to generate homozygous PxRyR lines. The inserted PxRyR sequence is not expressed in this line due to the absence of a Gal4 driver sequence. Instead, the endogenous DmRyR sequence (carried by the Cy (curly wing) allele marker) is expressed.


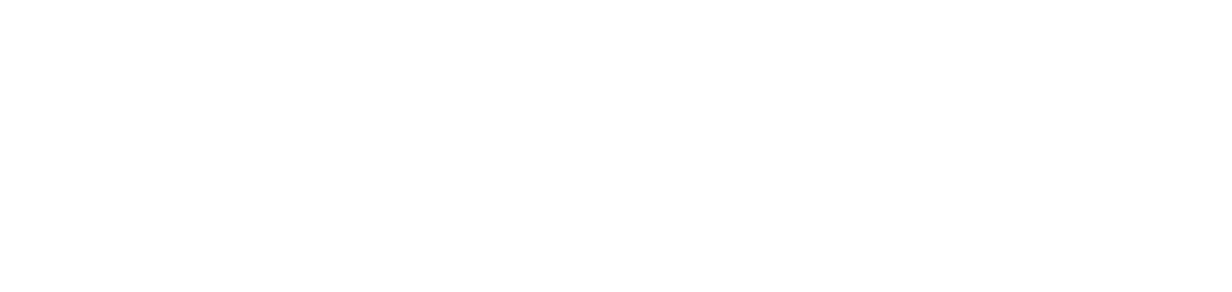


**
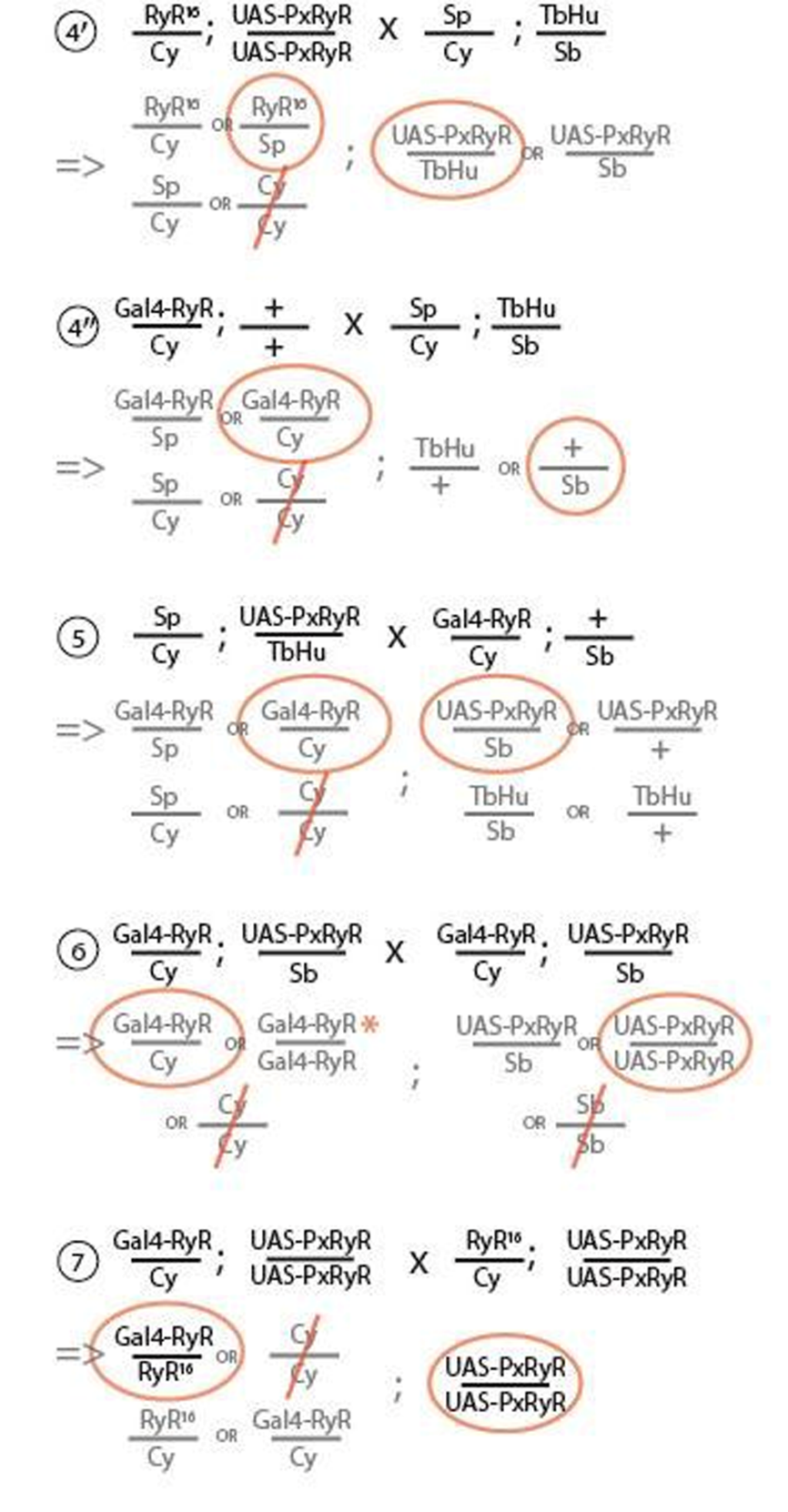
**

**Figure S5.** A series of crosses of the double marker line was used to recombine the inserted UAS-PxRyR into a line that also expresses the Gal4 promoter. Further crossing leads to the removal of the Cy marker, and with-it removal of the endogenous DmRyR expression. The asterisk (*) in panel (6) indicates the ‘stable line’ genotype that is obtained only at development temperatures below 17oC.

**
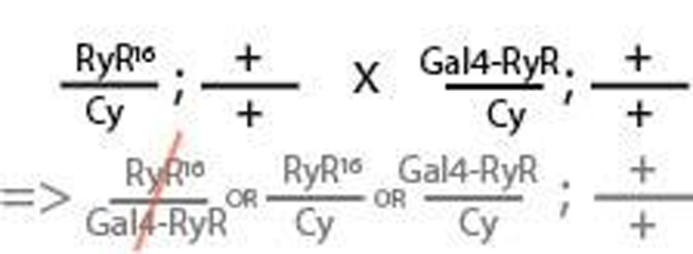
**

**Figure S6.** Attempted recombination of RyR^16^ and RyR-Gal4 null alleles into the same line. Flies fail to develop past larval stage L1.


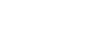

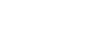


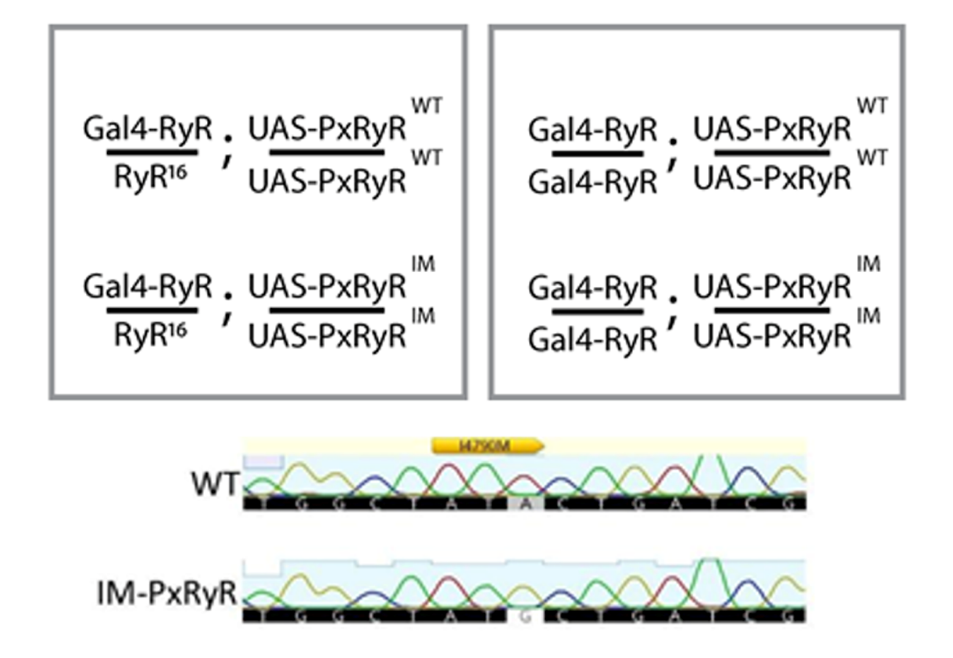


**Figure S7.** Drosophila lines generated. Stable lines (RH panel) were used during all experiments detailed, except for adult bioassays in which the RyR16 lines (LH panel) were used. Sequencing trace confirmation of successful integrations in the F2 generation (lower panel).
